# The biological effects of diagnostic cardiac imaging on chronically exposed physicians: the importance of being non-ionizing

**DOI:** 10.1186/1476-7120-2-25

**Published:** 2004-11-22

**Authors:** Maria Grazia Andreassi

**Affiliations:** 1 Laboratory of Cellular Biology and Genetics, CNR Institute of Clinical Physiology, Pisa, Italy

## Abstract

Ultrasounds and ionizing radiation are extensively used for diagnostic applications in the cardiology clinical practice. This paper reviewed the available information on occupational risk of the cardiologists who perform, every day, cardiac imaging procedures. At the moment, there are no consistent evidence that exposure to medical ultrasound is capable of inducing genetic effects, and representing a serious health hazard for clinical staff. In contrast, exposure to ionizing radiation may result in adverse health effect on clinical cardiologists. Although the current risk estimates are clouded by approximations and extrapolations, most data from cytogenetic studies have reported a detrimental effect on somatic DNA of professionally exposed personnel to chronic low doses of ionizing radiation. Since interventional cardiologists and electro-physiologists have the highest radiation exposure among health professionals, a major awareness is crucial for improving occupational protection. Furthermore, the use of a biological dosimeter could be a reliable tool for the risk quantification on an individual basis.

## Introduction

Over the last 30 years, medical cardiology imaging has rapidly grown, becoming an essential part of the cardiology clinical practice. Imaging procedures include conventional imaging tests such as echocardiography, radionuclide imaging, and angiography as well as a newer imaging techniques such as emission computed tomography and magnetic resonance imaging which promise to expand diagnostic capabilities [1]. These techniques widely differ not only for what concerns costs, availability and technical information, but they also differ in environmental and health hazards.

Many cardiac procedures can deliver high radiation doses to the clinical staff [2]. This exposure may represent a significant health risk, resulting in deleterious clinical implications which can affect not only the personnel involved, but also their progeny [3-5]. Unfortunately, many physicians are unfamiliar with radiation biology or the quantitative nature of the risks and, frequently, ultrasound and ionizing radiation risks are misunderstood [6-9]. The purpose of this paper is to discuss the published evidence on health effects of cardiac imaging procedures employing ultrasound and ionizing radiation.

## Ultrasound imaging

**Figure 1:**
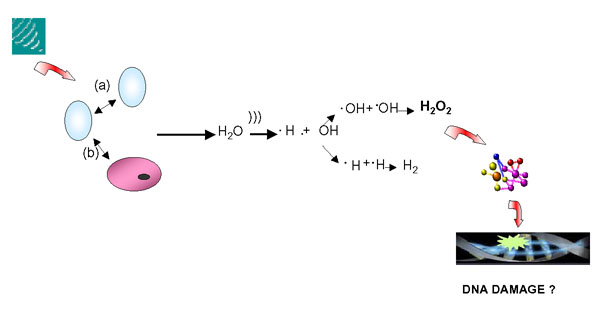
At high acoustic pressure, ultrasound is capable of causing rapid bubble which grow and collapse among them (a) and cells (b). This mechanism results in the production of sufficient energy to disrupt chemical bonds and produce reactive free radicals, that may interfere with DNA.

Ultrasound imaging, also called sonography, is a method of obtaining human body images through the use of high frequency sound waves. Ultrasounds are mechanical vibrations with frequencies above the human limit of audibility. The use of ultrasounds in order to obtain images for medical diagnostic purposes, typically employs frequencies ranging from 2 MHz to about 12 MHz [10]. Ultrasound does not use ionizing radiation, and it is the preferred image modality for monitoring both pregnant women and their embryos or fetus [10]. In contrast to ionizing radiation, which can damage biological materials by dislodging electrons from atoms and molecules, ultrasounds do not cause ionisation. They usually interact with human tissue primarily by generating heat, but also non-thermal effects which are ascribed to cavitation (i.e. micro-bubble) [11]. The process of cavitation includes ultrasounds mechanical effects which lead to hydrodynamic breaks of hydrogen bonds and oscillation of hydrogen ions, and chemical effects produced by the occurrence of free radicals in intercarionic space in the process of cavitation (Figure 1). Theoretically, these free radicals may interfere with DNA, causing chromosomal damage. Indeed, ultrasounds of diagnostic intensities induced detectable DNA damage in animal cells [12,13]. Currently, there is a body of studies on human DNA damage from exposure to therapeutic and diagnostic ultrasounds [14-20]. In particular, Stella et al. [15] reported that therapeutic ultrasound induce a significant increase in sister chromatid exchanges (SCEs) in human lymphocytes after treatment both *in vitro and in vivo*. In the same study, no increase in chromosomal aberrations was observed during and after ultrasound therapy [15]. Subsequently, some reports on human cells indicated that ultrasound was not able to induce SCEs or chromosomal damage (Table 1). Thus, there is at present no indication that exposure to medical ultrasound is capable of inducing genetic effects and representing a serious health hazard for clinical staff. However, very little information is available on the genetic effects of individuals occupationally exposed to chronic ultrasound. Medical staff can be exposed to hand-transmitted ultrasound waves in the work-place.

**Table 1:** Summary of studies on genetic effects of medical ultrasounds

**Author, Year (Ref)**	**Assay System**	**Endpoint**	**Exposure**	**Result**
Miller et al., 1983 (14)	Human lymphocytes exposed in vitro	SCE	2 MHz SPPA intensity 100 W/cm^2^	Negative
Stella et al., 1984 (15)	Human lymphocytes exposed in vitro	SCE CA	1 W/cm2; 0.860 MHz; for 40–160 sec	Positive/ Negative
Barnett et al., 1987 (16)	Human lymphocytes exposed in vitro	SCE	3.1 MHz SPPA intensities from 15 to 135 W/cm^2^.	Negative
Carrera P et al., 1990 (17)	Chorionic villi exposed in vitro Chorionic villi from exposed pregnant women	SCE	2 MHz at 1, 2, 3 h Diagnostic US for 20 min (in vivo exposure	Negative
Miller et al., 1991 (18)	Human lymphocytes from exposed patients	SCE	4 patients underwent therapeutic US 4 healthy persons underwent sham-therapeutic US	Negative
Martini et al., 1991 (19)	Lymphocyte and lymphoblastoid cells exposed in vitro	SCE	5 MHz for 20 sec, 1 min, 5 min, and 20 min	Negative
Sahin O et al., 2004 (20)	Human lymphocytes from exposed patients	MN	10 patients underwent 10 session of US therapy at 1 MHz for 10 min and 10 control subjects underwent sham-therapeutic US	Negative
Garaj-Vrhovac and Kopjar, 2000 (22)	Human lymphocytes from cardiologists working with Doppler ultrasound	CA SCE MN	Unit working with colour Doppler US (transducer frequencies 2.5–7.5 MHz. SPPA intensity 60–110 W/cm^2^.	Positive

SCE: sister-chromatid exchange; MN: Micronuclei; CA: Chromosomal aberrations; SPPA: Spatial Peak Pulse Average

Indeed, ultrasound sources do not transmit acoustic energy into air, and only low level ultrasound reaches medical personnel through handling of the probe [21]. Probably, occupational exposure to ultrasound occurs during training procedures [21]. In fact, medical personnel often apply diagnostic ultrasound to themselves during training or during technique demonstrations [21]. Consequently, ultrasound is not harmful like the other types and sources of radiation. However, a recent investigation indicated that medical personnel from a cardiology unit working with colour Doppler ultrasonic equipment had an increased genotoxic damage compared to the control subjects [22]. Therefore, this observation requires further studies in order to determine if chronic exposure to ultrasound might induce genotoxic effects.

## Ionizing radiation

Ionizing radiation is known to cause harm. High radiation doses tend to kill cells, while low doses tend to damage or alter the genetic code (DNA) of irradiated cells. The biological effects of ionizing radiation are divided into two categories: deterministic and stochastic effects. Deterministic effects, such as *erythema *or *cataract*, have a threshold dose below which the biological response is not observed [23-25]. Some interventional procedures with long screening times and multiple image acquisition (e.g. percutaneous coronary intervention, radio-frequency ablation, etc) may give rise to deterministic effects in both staff and patients [26,27].

A stochastic effect is a probabilistic event and there is no known threshold dose. The likelihood of inducing the effect, but not the severity, increases in relation to dose and may differ among individuals.

**Figure 2:**
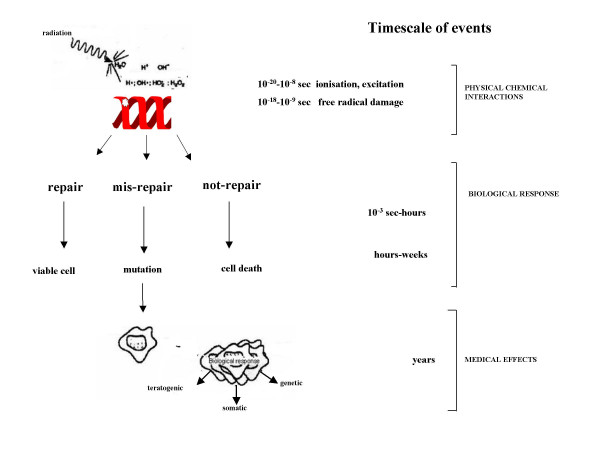
Radiation damage of DNA. Damaged DNA is screened through the process of DNA repair and mismatch correction. DNA lesions that escape repair, has the ability to produce mutations, which lead to the development and the progression of both cancer and human diseases even decades after exposure.

In fact, the effect of low doses of radiation -less than 50 mSv- do not cause an immediate problem to any body organ, but spread out over long periods of time after exposure. The biological effects are at DNA level and they may not be detected [23-25]. The cell has repair mechanisms against damage induced by radiation as well as by chemical carcinogens. Consequently, biological effects of low dose radiation on living cells may result in three outcomes: (1) injured or damaged cells repair themselves, resulting in no residual damage; (2) cells die; or (3) cells incorrectly repair themselves resulting in a biological change (Figure 2). Such biological changes include the development of cancer and genetic defects in the future children of exposed parents. At present, however, the effects of low-level exposure remain uncertain [28]. The associations between radiation exposure and the development of cancer are mostly based on populations exposed to relatively high levels of ionizing radiation (e.g., Japanese atomic bomb survivors). Since extraordinary large studies are required to quantify the risks of very low doses of radiation, it is unlikely that we will be able to precisely quantify cancer risk in human populations at doses below 10 mSv [28]. For instance, an epidemiological study of more than 5 million people would be needed to quantify the effect for a 10 -mSv dose or less [28]. Our inability to quantify risk does not, however, imply that this risk is negligible. Furthermore, the small (and often not so small) individual risk applied to a large number of individuals, and by protracted exposures, translates into a significant public health problem). As such, the international scientific community has adopted a prudent approach and acknowledged the fact that any level of exposure could potentially lead to biological effects. A linear, no-threshold dose response relationship is used by the IRCP in order to describe the relationship between radiation dose and the occurrence of cancer [29]. This dose-response model suggests that any increase in dose, no matter how small, results in an incremental increase in risk.

Genetic effects are the result of a mutation produced in the reproductive cells of an exposed individual that are passed on to their offspring. These effects may show up as birth defects or other conditions in the future children of the exposed individual and succeeding generation. Indeed, studies with laboratory animals have provided a large body of data on radiation-induced genetic effects [30]. Recently, these effects have been also observed in studies of people exposed to radiation from Chernobyl disaster, radiation workers and medical radiologists who have received doses of radiation [31-33]. However, no conclusive evidence exists yet [34,35].

## Radiation exposure to cardiologists

The use of radiation in medicine is the largest source of man-made radiation exposure. According to the latest estimation of the United Nations, an average of 2.4 mSv/year comes from natural sources [24]. In western countries, the exposure dose from medical radiation corresponds to 50 to 100% of the total natural radiation. In 1997, the German Federal Office for Radiation Protection reported 136 million x ray examinations and 4 million nuclear medicine diagnostic tests, resulting in a mean effective dose of 2.15 mSv per person per year [36]. Cardiac and interventional procedures account for a large percentage of nuclear and radiological examinations [36]. Of all radiological examinations, 28% are arteriographies and interventions. An additional 2% derive from chest X-rays and 37% from CT: many of them are cardiological referrals. Regarding nuclear medicine, 22% are cardiological scan. These percentages are likely higher now, since the use of cardiac and interventional procedures is increasing.

Cardiac ionizing procedures expose both patients and medical staff to the highest radiation levels in diagnostic radiology, and recently, as the number of diagnostic and interventional cardiac catheterisation procedures has greatly increased, serious radiation induced skin injuries and an excess of cataract development have been reported in exposed staff [37-39]. Furthermore, it has been suggested that fluoroscopic procedures may be a health hazard and increase the risk for brain tumours in interventional cardiologists [40].

Today, interventional cardiologists represent, indeed, the most important group of exposed among professionally exposed physicians [41,42].

As known, the limit on effective dose for exposed workers should be 100 mSv in a consecutive five year period, subject to a maximum effective dose of 50 mSv in any single year. Radiation dose limits to adult occupational workers provided by the International Commission on Radiological Protection (ICRP) are shown in table 2.

**Table 2:** Recommended occupational dose limits by International Commission on Radiological Protection (ICRP).

**TISSUE INJURY**	**OCCUPATIONAL DOSE LIMITS/YEAR**	
whole body	20 mSv	2 rem

Lens of the eye	150 mSv	15 rem

Skin, hands, feet, and other organs	500 mSv	50 rem

As a matter of fact, the head dose sustained by cardiologists may reach 60 mSv per year, and may in some cases exceed the occupational limit of 150 mSv per year recommended for the lens of the eye [41].

However, the correlation between occupational doses and staff radiological risks is not simple, and it is very dependent on equipment, the specialist, and protocols followed throughout the procedure [43]. Many factors can influence occupational doses for the same radiation dose imparted during cardiac procedure. One of the most important factors is that protection tools are available in catheterisation laboratories and are appropriately used [43]. In addition, another likely reason is a lack of knowledge, information and training in radiation protection [43].

Importantly, a recent survey showed that that most of cardiologists do not correctly evaluate the dose exposure, the medico-legal regulation, the environmental impact and individual bio-risks of the radiological investigations [9]. As shown in table 3, this surprising lack of knowledge of both dose and clinical risk of commonly performed ionising test examinations, is not at all restricted to cardiologists, and seems to be democratically spread across all specialties – from surgeons to orthopaedics, to paediatricians [6-9].

**Table 3:** Doctors' knowledge of radiation dose and risk for medical ionising testing

**Author, year (Ref)**	**Physicians**	**Radiological Awareness Evaluation**	**Results**
Shiralkar S et al., 2003 (6)	British physicians	Radiation doses for common radiological investigations.	97% of doctors underestimates dose. 5% believes that US use ionising radiation. 8% believes thatMRI use ionising radiation.

Finestone A et al., 2003 (7)	Istraeli orthopaedists	Mortality risk of radiation-induced carcinoma from bone scan scintigraphy	Mortality risk was identified correctly by less than 5% of respondents.

Lee CI et al., 2004 (8)	Emergency department (ED), physicians and radiologists	Radiation dose and possible risks associated with CT scan	Almost all doctors were unable to accurately estimate the dose. Only 9% ED physicians believed that there was increased risk.

Correia MJ et al., 2005 (9)	Adult and paediatric cardiologists	Environmental impact, individual bio-risks, dose exposure and medico-legal regulation of medical ionising testing	Only 11%, 5%, 29% and 42% of physicians correctly identified environmental impact, individual bio-risks, dose exposure and legal regulation, respectively.

CT = computed tomography; MRI = magnetic resonance imaging; US = ultrasound

Probably, this unawareness has its root in the difficult perception of a long-term risk associated to radiation exposure. In particular, the perception of cancer risk, which can have a latency period of many years after exposure, is often elusive. Furthermore, the exact risk at very low doses to a specific individual is further complicated by many factors, such as carcinogenic agents in our environment, cigarette smoke, diet and genetic background.

However, a recent study has estimated that from 0.6% to 3% of all cancers are due to medical X-rays [44]. These figures are impressive but may largely underestimate the true risk, since they are referred to radiological data concerning the 1991–1996. Taking into account current radiological activities, medical radiation is likely to account for at least 20% of cancer in developed countries [45].

With regard to occupational exposure for radiologists and radiotherapists, available epidemiological studies have been recently reviewed by Yoshinaga et al [46]. An excess risk of leukaemia associated with occupational radiation was found among early workers employed before 1950, when radiation exposures were high. In addition, several studies provided evidence of a radiation effect for breast and skin cancer. To date, there is no clear evidence of an increased cancer risk in medical radiation workers exposed to current levels of radiation doses. However, given a relatively short period of time for which the most recent workers have been followed up and in view of the increasing uses of radiation in modern medical practices, it is important to continue to monitor the health status of medical radiation workers [46].

To the fatal cancer risk, one must add the risk of non-fatal cancer and major genetic damage transmitted to the offspring. It is relevant to underline that the long-term damage may not include only cancer but also other major degenerative diseases, including atherosclerosis [47,48]. However, it is important to realize that many difficulties are involved in designing epidemiological studies that can accurately measure the increases in health effects due to low exposures to radiation as compared to the normal rate of cancer. Studies with very large sample size are required in order to quantify the risks of very low doses of radiation. An alternative strategy could be based on the measure of biological effects by using *biomarkers *as predictors of delayed health outcomes [49].

## Biomarkers in the assessment of radiation exposure

**Figure 3:**
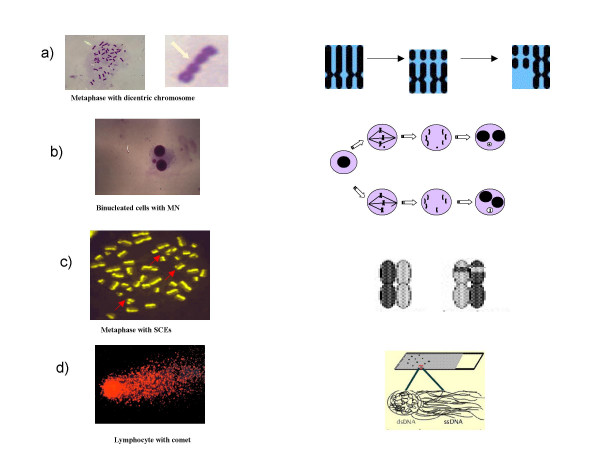
Biomarkers of DNA damage in human lymphocytes: a) Structural chromosomal aberrations (CA) are typical of cancer cells, probably as a manifestation of genetic instability. b) Micronuclei (MN) can originate from chromosome breaks or whole chromosomes that fail to engage with the mitotic spindle when the cell divides. Therefore, the micronucleus test can be considered just as a real "biological dosimeter" for evaluating both numerical and structural chromosome aberrations. c) Sister chromatid exchanges (SCEs) represent symmetrical exchanges between sister chromatids; generally they do not result in chromosomal alterations of the genetic information. c) The Comet assay is an especially sensitive method for detecting DNA single-strand breaks and oxidative DNA damage in individual cells. The entity of the DNA damage is proportional to the length of the comet.

Damage to deoxyribonucleic acid (DNA), which carries the genetic information in chromosomes in the cell nucleus, is considered to be the main initiating event by which radiation damage to cells results in the development of cancer and hereditary disease. Four biomarkers (Figure 3) -analysis of structural chromosome aberrations, micronucleus assay, sister chromatid exchange analysis and comet assay- in peripheral lymphocytes are currently employed in order to study human exposure to environmental carcinogens [50]. Among these, the test of chromosomal aberrations in peripheral blood lymphocytes has the most abundant literature validating that a high frequency of chromosomal breakage is a strong predictor of cancer risk in healthy subjects [51,52].

During the last years, the micronucleus assay has become popular since it is fast and inexpensive, and it is considered to be a "biological dosimeter" for exposure to ionizing radiation [53].

**Figure 4:**
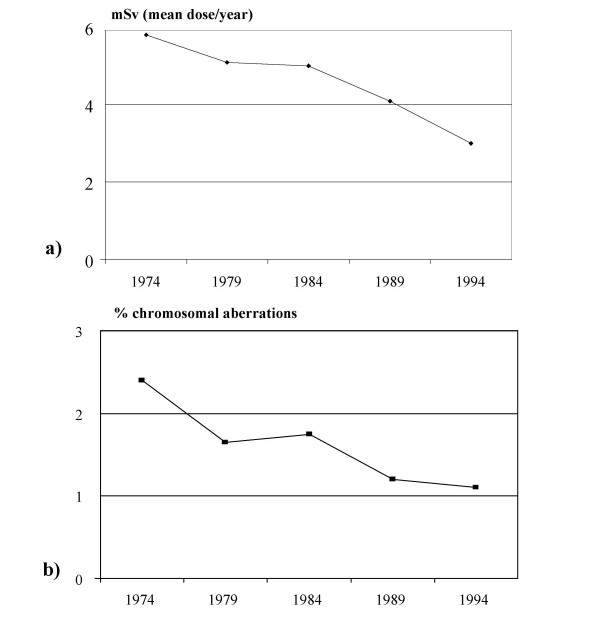
a) Decrease in exposure to ionizing radiation in hospital radiologists over the most recent decades and b) a similar time-related reduction in the frequency of chromosome-type aberrations (redrawn from ref. 58)

The importance of cytogenetic study of peripheral lymphocytes in subjects exposed to ionizing radiation has been reported for more than 20 years, especially in radiologists [54-68]. The available evidence suggests that chronic exposure to low dose radiation has a genotoxic effect on somatic DNA of professionally exposed workers (Table 4). This effect seems to be cumulative over time, although the majority of these studies failed to establish a dose-effect relationship for low doses. The absence of increase of somatic DNA damage in relation to the dose might be explained by various factors. Dosimetry records may underestimate the real dose exposure if the badges are not properly worn. The potential combined effect of other genotoxic exposures would also induce DNA damage, enhancing the effect of radiation exposure [63]. Moreover, genetic susceptibility may account for the inter-individual differences to radiation sensitivity. Such possible susceptibility may recognize sources of variability (genetic polymorphism) in people's DNA repair gene sequence [69]. However, it is interesting to underline that, in a group of radiologists, it has been documented an important parallelism between the decrease of the exposure to ionizing radiation in the hospitals and a reduction in the frequency of chromosome aberrations over the most recent decades [58] (Figure 4). This decrease was the result of an efficient protection policy among radiologists. Unfortunately, this is not the case for invasive cardiologists who need to know very well both the long-term risks and the doses involved in the large amount of examinations they prescribe and/or perform every day [40,41].

**Table 4:** Cytogenetic studies in hospital workers

**Author, year (ref)**	**Exposed Subjects, n**	**Non-exposed Subjects, n**	**Endpoint**	**Results**	**Exposure**	**Correlation with dose (Yes/No)**
Bigatti et al, 1988, (54)	63 (physicians, nurses and technicians)	30 (ward nurses and office personnel)	CA	Positive	< legal limit.	No
Barquinero et al, 1993, (55)	26 (hospital workers)	10 (healthy individuals)	CA	Positive	1.6–42.71 mSv	No
Paz-y-Mino et al, 1995, (56)	10 (hospital workers)	10 (healthy individuals)	CA	Positive	1.84 mSv/year.	No
Vera et al, 1997, (57)	20 (medical staff working at an X-ray department)	20 (general population)	CA MN	Positive	<25 mSv/year.	No (Major DNA damage in subjects exposed to both ultrasound and X-ray)
Bonassi et al., 1997, (58)	871 (hospital workers from 4 laboratories)	617 (healthy individuals)	CA	Positive	Available only partially and variable.	Yes/No
Rozgaj et al, 1999, (59)	483 (radiologists, pneumologists, technicians)	160 (healthy individuals)	CA	Positive	<20 mSv/year	No
Undeger et al., 1999, (60)	30 (technicians)	30 (nurses, technicians, office personnel)	Comet	Positive	50 mSv/ year.	No
Cardoso et al, 2001, (61)	8 (workers in X-rays, radiotherapy and nuclear medicine sectors)	8 (healthy individuals)	CA MN SCE	Positive	63.2 mSv/life	No
Maluf et al, 2001, (62)	22 (hospital workers)	22 (non-exposed workers)	MN Comet	Positive	0.2 – 121. mSv	No
Maffei et al, 2002, (63)	37 (physicians, technicians)	37 (non-exposed workers	MN	Negative/ Positive	35 mSv /life	No
Bozkurt et al, 2003, (64)	16 (nuclear medicine)	16 (non-exposed physicians)	SCE	Positive	3.39 mSv/year.	Yes
Garaj-Vrhovac and Kopjar, 2003, (65)	50 (physicians, 25 technicians, 10 nurses)	50 (healthy students and office employees)	Comet	Positive	0–8.5 mSv/year.	No
Maffei et al, 2004, (66)	34 (physicians, technicians)	35 (non-exposed workers)	CA	Positive	1.81–141.77 mSv/life.	Yes
Zakeri et al., 2004, (67)	71 (cardiologists, nurses and technicians)	36 (healthy individuals)	CA MN	Positive	3.0 mSv/year	No
Andreassi et al, 2004, (67)	31 interventional cardiologists	31 clinical cardiologists	MN	Positive	4 mSv/year	No

SCE: sister-chromatid exchange; MN: Micronuclei; CA: Chromosomal aberrations;

As matter of fact, our results and a recent monitoring of personnel working in angiocardiography laboratories in Iranian Hospitals showed a high frequency of chromosome aberrations in cardiologists s and technicians compared to unexposed subjects [68,69].

Taken together, these evidences highlight that the use of a biological dosimeter could complement the data obtained by physical dosimetry and reduce the uncertainties of low-dose radiation risk assessment [70]. The analysis of chromosome aberrations is the gold standard endpoint for radiation biological dosimetry. Limitations and strengths on biodosimetry have been fully discussed in the IAEA Report 405 [70]. A possible limitation is the response to high radiation dose (> 4 Sv) where cell death and delays in progression through the cycle represents a pitfall for estimation of acute irradiation particularly when non-uniform or partial body irradiation have occurred.

Moreover, the method is laborious, time consuming and requires expert skills. Scoring of micronuclei has been proposed as an alternative to conventional chromosome aberrations analysis, being more sensitive and faster [71]. Although micronuclei method has been improved, inter-laboratories discrepancies have emphasized the need for better standardization [53].

**Figure 5:**
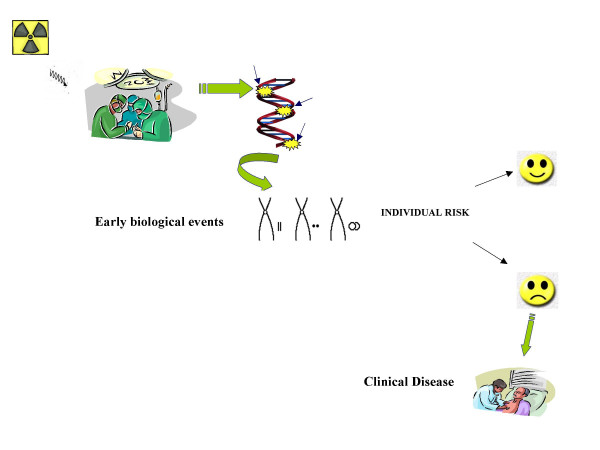
Illustration of potential use of biomarkers as early predictors of clinical disease. The evaluation of genetic effects such as chromosomal damage could be used to anticipate delayed health outcomes, providing a greater potential for preventive measures.

However, in many countries the application of cytogenetic dosimetry has yet medical-legal recognition, and it is complementary to physical dosimetry. On the other hand, the usefulness of biomarkers as early biological effects, with special concern for the prediction of cancer, has been recently emphasized [72]. Therefore, the application of biodosimetry- that measures true cellular injury resulting from that radiation- could greatly enhance health risk, identifying susceptible individuals and enhancing the possibility of preventive measures, especially in occupational settings with a high volume of radiological activities (Figure 5).

## Conclusion

Occupational exposure can occur in cardiological procedures which employ ultrasound and ionizing radiation. Today, there are no consistent adverse biological effects on operators caused by exposures to ultrasound. However, it is clearly necessary to continually monitor both the potential risks and safety of ultrasound exposure. In contrast, exposure to ionizing radiation may result in adverse health effect on both cardiologists directly and on their progeny. Although the current risk estimates are clouded by approximations and extrapolations, most data from cytogenetic studies have reported an enhanced DNA damage in hospital workers exposed to chronic low doses of ionizing radiation. The occupational dose of interventional cardiologists, and electrophysiologists tend to be higher compared to other medical specialists as a result of the recent increasing use of interventional techniques. On the other hand, physicians are dramatically unaware of dose, long-term risks and populations health impact caused by the use of medical ionizing radiation. Thus, a major awareness appears to be crucial in order to improve both one's knowledge on the appropriateness of protective tools and also in trying to reduce the number of unnecessary procedures. The use of a biological dosimeter could be a reliable tool for risk quantification on an individual basis.

## References

[B1] Higgins CB (2000). Cardiac imaging. Radiology.

[B2] International Commission on Radiological Protection (ICRP) (2001). Radiation and your patient: a guide for medical practitioners. A web module produced by Committee 3 of the ICRP.

[B3] European Commission. Radiation protection 118 Referral guidelines for imaging. http://europa.eu.int/comm/environment/radprot/118/rp-118-en.pdf.

[B4] Council Directive 97/43/Euratom of 30 June 1997 on health protection of individuals against the dangers of ionizing radiation in relation to medical exposure, and repealing Directive 84/466/Euratom. Official Journal of the European Communities.

[B5] Picano E (2004). Sustainability of medical imaging. Education and Debate. BMJ.

[B6] Shiralkar S, Rennie A, Snow M, Galland RB, Lewis MH, Gower-Thomas K (2003). Doctors' knowledge of radiation exposure: questionnaire study. BMJ.

[B7] Finestone A, Schlesinger T, Amir H, Richter E, Milgrom C (2003). Do physicians correctly estimate radiation risks from medical imaging?. Arch Environ Health.

[B8] Lee CI, Haims AH, Monico EP, Brink JA, Forman HP (2004). Diagnostic CT scans: assessment of patient, physician, and radiologist awareness of radiation dose and possible risks. Radiology.

[B9] Correia MJ, Hellies A, Andreassi MG, Ghelarducci B, Picano E (2005). Lack of Radiological Awareness in a Tertiary Care Cardiological Centre. Int J Cardiol.

[B10] Nyborg WL (2001). Biological effects of ultrasound: development of safety guidelines. Part II: general review. Ultrasound Med Biol.

[B11] American Institute of Ultrasound in Medicine (Bioeffects Committee) (1988). Bioeffects considerations for the safety of diagnostic ultrasound. American Institute of Ultrasound in Medicine. J Ultrasound Med.

[B12] Liebeskind D, Bases R, Elequin F, Neubort S, Leifer R, Goldberg R, Koenigsberg M (1979). Diagnostic ultrasound: effects on the DNA and growth patterns of animal cells. Radiology.

[B13] Fuciarelli AF, Sisk EC, Thomas RM, Miller DL (1995). Induction of base damage in DNA solutions by ultrasonic cavitation. Free Radic Biol Me.

[B14] Miller MW, Wolff S, Filly R, Cox C, Carstensen EL (1983). Absence of an effect of diagnostic ultrasound on sister-chromatid exchange induction in human lymphocytes in vitro. Mutat Res.

[B15] Stella M, Trevisan L, Montaldi A, Zaccaria G, Rossi G, Bianchi V, Levis AG (1984). Related Induction of sister-chromatid exchanges in human lymphocytes exposed in vitro and in vivo to therapeutic ultrasound. Mutat Res.

[B16] Barnett SB, Barnstable SM, Kossoff G (1987). Sister chromatid exchange frequency in human lymphocytes after long duration exposure to pulsed ultrasound. J Ultrasound Med.

[B17] Carrera P, Orsini S, Terzoli G, Brambati B, Simoni G (1990). Sister chromatid exchanges in first-trimester chorionic villi after in vivo and in vitro exposure to diagnostic ultrasound. Prenat Diagn.

[B18] Miller MW, Azadniv M, Cox C, Miller WM (1991). Lack of induced increase in sister chromatid exchanges in human lymphocytes exposed to in vivo therapeutic ultrasound. Ultrasound Med Biol.

[B19] Martin AO, Madsen EL, Dyer AR, White L, Bouck NP, Sabbagha RE, Hermanoff M, Chen JM, Ludtke LJ (1991). Sister chromatid exchange analysis of human cells exposed to diagnostic levels of ultrasound. J Ultrasound Med.

[B20] Sahin O, Donmez-Altuntas H, Hizmetli S, Hamurcu Z, Imamoglu N (2004). Investigation of genotoxic effect of ultrasound in cases receiving therapeutic ultrasound by using micronucleus method. Ultrasound Med Biol.

[B21] Nyborg WL (1996). Human Exposure to ultrasound. Appl Occup Environ Hygn.

[B22] Garaj-Vrhovac V, Kopjar N (2000). Cytogenetic monitoring of cardiology unit hospital workers exposed to Doppler ultrasound. J Appl Toxicol.

[B23] Beir V (1990). Health effects of exposure to low levels of ionizing radiation. Prepared by the Committee on the Biological Effects of Ionizing Radiation, National Research Council, Washington, DC.

[B24] Report of the United States Nations Scientific Committee on the Effects of Atomic Radiation to the General Assembly (2001). Annex G: Biological effects at low radiation doses. USCEAR.

[B25] Hall EJ (2000). Radiobiology for the Radiologist.

[B26] International Commission on Radiological Protection. ICRP Publication 59 (1991). The biological basis for dose limitation in the skin. Annals of the ICRP.

[B27] National Radiological Protection Board (1996). Risk from deterministic effects of ionizing radiation. Documents of the National Radiological Protection Board 7.

[B28] Brenner DJ, Doll R, Goodhead DT, Hall EJ, Land CE, Little JB, Lubin JH, Preston DL, Preston RJ, Puskin JS, Ron E, Sachs RK, Samet JM, Setlow RB, Zaider M (2003). Cancer risks attributable to low doses of ionizing radiation: assessing what we really know. Proc Natl Acad Sci USA.

[B29] National Council on Radiation Protection and Measurements (2001). Evaluation of the Linear-Nonthreshold Dose-Response Model for Ionizing Radiation. NCRP, Bethesda.

[B30] Dubrova YE (2003). Long-term genetic effects of radiation exposure. Mutat Res.

[B31] Gardner MJ, Snee MP, Hall AJ, Powell CA, Downes S, Terrell JD (1990). Results of case-control study of leukaemia and lymphoma among young people near Sellafield nuclear plant in West Cumbria. BMJ.

[B32] Hama Y, Uematsu M, Sakurai Y, Kusano S (2001). Sex ratio in the offspring of male radiologists. Acad Radiol.

[B33] Dubrova YE, Grant G, Chumak AA, Stezhka VA, Karakasian AN (2002). Elevated minisatellite mutation rate in the post-chernobyl families from ukraine. Am J Hum Genet.

[B34] Kiuru A, Auvinen A, Luokkamaki M, Makkonen K, Veidebaum T, Tekkel M, Rahu M, Hakulinen T, Servomaa K, Rytomaa T, Mustonen R (2003). Hereditary minisatellite mutations among the offspring of Estonian Chernobyl cleanup workers. Radiat Res.

[B35] Slebos RJ, Little RE, Umbach DM, Antipkin Y, Zadaorozhnaja TD, Mendel NA, Sommer CA, Conway K, Parrish E, Gulino S, Taylor JA (2004). Mini-and microsatellite mutations in children from Chernobyl accident cleanup workers. Mutat Res.

[B36] Regulla D, Griebel J, Nosske D, Bauer B, Brix G (2003). Acquisition and assessment of patient exposure in diagnostic radiology and nuclear medicine. Z Med Phys.

[B37] Renaud L (1992). A 5-year follow up of the radiation exposure to in-room personnel during cardiac catheterization. Health Phys.

[B38] Vano E, Gonzalez L, Beneytez F, Moreno F (1998). Lens injuries induced by occupational exposure in non-optimised interventional radiology laboratories. Br J Radiol.

[B39] McKetty MH (1996). Study of radiation doses to personnel in a cardiac catheterisation laboratory. Health Phys.

[B40] Finkelstein MM (1998). Is brain cancer an occupational disease of cardiologists?. Can J Cardiol.

[B41] Vano E (2003). Radiation exposure to cardiologists: how it could be reduced. Heart.

[B42] Delichas M, Psarrakos K, Molyvda-Athanassopoulou E, Giannoglou G, Sioundas A, Hatziioannou K, Papanastassiou E (2003). Radiation exposure to cardiologists performing interventional cardiology procedures. Eur J Radiol.

[B43] Kuon E, Birkel J, Schmitt M, Dahm JB (2003). Radiation exposure benefit of a lead cap in invasive cardiology. Heart.

[B44] Berrington De Gonzalez A, Darby S (2004). Risk of cancer from diagnostic X-rays: estimates for the UK and 14 other countries. Lancet.

[B45] Picano E (2004). Risk of Cancer from diagnostic X-rays: impressive but underestimated. Lancet.

[B46] Yoshinaga S, Mabuchi K, Sigurdson AJ, Doody MM, Ron E (2004). Cancer risks among radiologists and radiologic technologists: review of epidemiologic studies. Radiology.

[B47] Gofman JW (1999). Radiation from medical procedures in the pathogenesis of cancer and ischemic heart disease: dose-response studies with physicians per 100,000 population, San Francisco. Committee for Nuclear Responsibility Books, Available at UCSF Med Library The Exec Summary online.

[B48] Preston DL, Shimizu Y, Pierce DA, Suyama A, Mabuchi K (2003). Studies of mortality of atomic bomb survivors. Report 13: Solid cancer and noncancer disease mortality: 1950–1997. Radiat Res.

[B49] Bonassi S (1999). Combining environmental exposure and genetic effect measurements in health outcome assessment. Mutat Res.

[B50] Pavanello S, Clonfero E (2000). Biological indicators of genotoxic risk and metabolic polymorphisms. Mutat Res.

[B51] Hagmar L, Bonassi S, Stromberg U, Brogger A, Knudsen LS, Norppa H, Reuterwall C, European Study Group on Cytogenetic Biomarkers and Health (1998). Chromosomal aberrations in lymphocytes predict human cancer: a report from the European Study Group on Cytogenetic Biomarkers and Health (ESCH). Cancer Res.

[B52] Hagmar L, Stromberg U, Bonassi S, Hansteen IL, Knudsen LE, Lindholm C, Norppa H (2004). Impact of types of lymphocyte chromosomal aberrations on human cancer risk: results from Nordic and Italian cohorts. Cancer Res.

[B53] Fenech M (2002). Biomarkers of genetic damage for cancer epidemiology. Toxicology.

[B54] Bigatti P, Lamberti L, Ardito G, Armellino F (1988). Cytogenetic monitoring of hospital workers exposed to low-level ionizing radiation. Mutat Res.

[B55] Barquinero JF, Barrios L, Caballin MR, Miro R, Ribas M, Subias A, Egozcue J (1993). Cytogenetic analysis of lymphocytes from hospital workers occupationally exposed to low levels of ionizing radiation. Mutat Res.

[B56] Paz-y-Mino C, Leone PE, Chavez M, Bustamante G, Cordova A, Gutierrez S, Penaherrera MS, Sanchez ME (1995). Follow up study of chromosome aberrations in lymphocytes in hospital workers occupationally exposed to low levels of ionizing radiation. Mutat Res.

[B57] Vera GV, Aleksandra F, Dragan K, Andrija H (1997). Assessment of genome damage in occupational exposure to ionizing radiation and ultrasound. Mutat Res.

[B58] Bonassi S, Forni A, Bigatti P, Canevarollo N, De Ferrari M, Lando C, Padovani P, Bevegni M, Stella M, Vecchio D, Puntoni R (1997). Chromosome aberrations in hospital workers: evidence from surveillance studies in Italy (1963–1993). Am J Ind Med.

[B59] Rozgaj R, Kasuba V, Sentija K, Prlic I (1999). Radiation-induced chromosomal aberrations and haematological alterations in hospital workers. Occup Med.

[B60] Undeger U, Zorlu AF, Basaran N (1999). Use of the alkaline comet assay to monitor DNA damage in technicians exposed to low-dose radiation. J Occup Environ Med.

[B61] Cardoso RS, Takahashi-Hyodo S, Peitl P, Ghilardi-Neto T, Sakamoto-Hojo ET (2001). Evaluation of chromosomal aberrations, micronuclei, and sister chromatid exchanges in hospital workers chronically exposed to ionizing radiation. Teratog Carcinog Mutagen.

[B62] Maluf SW, Passos DF, Bacelar A, Speit G, Erdtmann B (2001). Assessment of DNA damage in lymphocytes of workers exposed to X-radiation using the micronucleus test and the comet assay. Environ Mol Mutagen.

[B63] Maffei F, Angelini S, Forti GC, Lodi V, Violante FS, Mattioli S, Hrelia P (2002). Micronuclei frequencies in hospital workers occupationally exposed to low levels of ionizing radiation: influence of smoking status and other factors. Mutagenesis.

[B64] Bozkurt G, Yuksel M, Karabogaz G, Sut N, Savran FO, Palanduz S, Yigitbasi ON, Algunes C (2003). Sister chromatid exchange in lymphocytes of nuclear medicine physicians. Mutat Res.

[B65] Graj-Vrhovac Vera, Kopjar N (2003). The alkaline Comet assay as biomarker in assessment of DNA damage in medical personnel occupationally exposed to ionizing radiation. Mutagenesis.

[B66] Maffei F, Angelini S, Forti GC, Violante FS, Lodi V, Mattioli S, Hrelia P (2004). Spectrum of chromosomal aberrations in peripheral lymphocytes of hospital workers occupationally exposed to low doses of ionizing radiation. Mutat Res.

[B67] Zakeri F, Assaei RG (2004). Cytogenetic monitoring of personnel working in angiocardiography laboratories in Iran hospitals. Mutat Res.

[B68] Andreassi MG, Joksic G, Manfredi S, Alavantic D, Cioppa A, Botto N, Hellies A, Ostojic M, Rubino P, Picano E (2004). omatic DNA damage in interventional cardiologists. Eur Heart J.

[B69] Aka P, Mateuca R, Buchet JP, Thierens H, Kirsch-Volders M (2004). Are genetic polymorphisms in OGG1, XRCC1 and XRCC3 genes predictive for the DNA strand break repair phenotype and genotoxicity in workers exposed to low dose ionising radiations?. Mutat Res.

[B70] International Atomic Energy Agency (IAEA) (2001). Cytogenetic analysis for Radiation Dose Assessment. Technical Report.

[B71] Fenech M, Gledhill BL, Mauro F, Ed (1981). Optimisation of micronucleus assays for biological dosimetry. in New Horizons in Biological Dosimetry (Wiley, New York).

[B72] Bonassi S, Au WW (2002). Biomarkers in molecular epidemiology studies for health risk prediction. Mutat Res.

